# Involvement of PKCε in FSH-induced connexin43 phosphorylation and oocyte maturation in mouse

**DOI:** 10.1242/bio.034678

**Published:** 2018-07-30

**Authors:** Han Cai, Bingying Liu, Tingting Yang, Yi Yang, Jinrui Xu, Zhiqing Wei, Guangcun Deng, Gang Ning, Junxia Li, Jing Wen, Wei Liu, Zhangli Ni, Yuzhen Ma, Meijia Zhang, Bo Zhou, Guoliang Xia, Hong Ouyang, Chao Wang

**Affiliations:** 1 State Key Laboratory of Agrobiotechnology and Department of Animal Physiology, College of Biological Sciences, China Agricultural University, Beijing 100193, China; 2 Key Laboratory of Ministry of Education for Conservation and Utilization of Special Biological Resources in the Western China, College of Life Science, Ningxia University, Yinchuan, Ningxia 750021, China; 3 Department of Obstetrics and Gynecology, Center of Reproductive Medicine, Inner Mongolia People's Hospital, Hohhot, Inner Mongolia 010017, China; 4 State Key Laboratory of Ophthalmology, Zhongshan Ophthalmic Center, Sun Yat-sen University, Guangzhou 510060, China

**Keywords:** Connexin43, cAMP, PKC epsilon, Mouse, Oocyte maturation

## Abstract

Gap junctions (GJs) are indispensable for communication between cumulus cells (CCs) and oocytes in coordinating the gonadotropin-induced meiotic maturation of oocytes. Of all proteins that constitute GJs, phosphorylated connexin43 (pCx43) is vital for mediating the actions of gonadotropins. In this study, the mechanism of Cx43 phosphorylation in response to follicle stimulating hormone (FSH) stimulation was examined using an *in vitro* model of mouse cumulus-oocyte complexes (COCs). The results confirmed that Cx43 phosphorylation occurred twice during FSH treatment. Importantly, the second Cx43 phosphorylation was closely related to cAMP level reduction within oocytes, which initiated oocyte maturation. Exploration of the underlying mechanism revealed that the CC-specific protein kinase C ε (PKCε) level was upregulated by FSH stimulation. PKCε was a kinase downstream from mitogen-activated protein kinase (MAPK) and was responsible for Cx43 phosphorylation. Interestingly, MAPK was involved in both Cx43 phosphorylation processes, while PKCε was only involved in the second. In conclusion, PKCε-mediated MAPK signals might contribute to Cx43 phosphorylation in CCs during FSH-induced oocyte meiotic resumption. Our findings contribute to a better understanding of the molecular regulation mechanism of oocyte maturation in response to FSH *in vitro*.

## Abstract (precis)

**Summary:** This research clarifies the important role of PKCε in mediating MAPK action on CX43 phosphorylation during FSH-induced oocyte maturation *in vitro*, and further explores the regulation mechanism of oocyte maturation.

## INTRODUCTION

In mammals, the endocrine control of meiotic arrest and resumption rests on a network of extracellular and intracellular molecular interactions within follicles (Zhang et al., 2011). Gap junctions (GJs) are intercellular channels that directly link adjacent cells to allow for the exchange of small molecules (Harris, 2007). The building blocks of GJs are connexins (Cxs), which are a family of approximately 20 proteins that form GJ channels for assisting intercellular communication (Winterhager and Kidder, 2015). GJs are essential for interactions between somatic cells and oocytes in follicles. With GJs, somatic cells can pass nutrients, e.g. amino acids and glucose, to support the growing metabolic requirements of an oocyte, ions to regulate the pH within the oocyte, or inhibitory signals, such as cyclic adenosine monophosphate (cAMP) and cyclic guanosine monophosphate (cGMP) from cumulus cells (CCs), to maintain meiotic arrest (Fagbohun and Downs, 1991; Downs, 1995; Conti et al., 2002; Thomas et al., 2004; Mehlmann, 2005; Kidder and Vanderhyden, 2010; Zhang et al., 2010). However, the functions of GJs during oocyte meiotic resumption are far from well understood.

Different types of Cxs are involved in the communication between the somatic and oocytic compartments of a follicle at specific stages. We have shown that the nonspecific blocking of GJ communication (GJC) in the fetal mouse ovary suppresses primordial folliculogenesis (Wen et al., 2009) and that GJs are essential for the assembly of primordial follicles in a narrow time window by cross-talk with Notch signaling in mouse ovaries (Teng et al., 2015)**.** In mature ovaries, granulosa cells (GCs) are interconnected via Cx43 and Cx45, while connection to the oocyte via the surrounding CCs involves Cx37 and Cx43 (Feuerstein et al., 2007; Zuccotti et al., 2011). Importantly, Cx43 phosphorylation is associated with GJ closure and possibly blocks inhibitory signals to the oocyte, which appears sufficient to induce oocyte meiotic resumption (Norris et al., 2008). Studies in rodent oocytes have also suggested that GJ disconnection, especially that involving Cx43, precedes meiotic resumption (Norris et al., 2008; Zhang et al., 2010). However, the precise relationship between the loss of this coupling and the timely resumption of oocyte meiosis needs further study.

During luteinizing hormone (LH)-induced oocyte meiotic resumption *in vivo*, epidermal growth factor receptor (EGFR) trans-activation includes the mitogen-activated protein kinase (MAPK)-dependent phosphorylation of Cx43 on specific serine residues. In turn, this reduces the flux of cGMP and initiates oocyte meiosis (Norris et al., 2008, 2010; Zhang et al., 2010). However, multiple signaling cascades downstream of the LH receptor (LHR), including the EGFR and possibly the PKC signaling pathways, may also mediate MAPK activation (Woods and Johnson, 2007). Notably, in clinical practice, follicle-stimulating hormone (FSH) is applied in the *in vitro* culture procedures of human and livestock oocytes because FSH alone is sufficient to stimulate not only cumulus expansion in isolated cumulus-oocyte complexes (COCs), but also oocyte meiotic resumption *in vitro* (Ali and Sirard, 2002; Blondin et al., 2002; Kawamura et al., 2011). FSH-induced oocyte maturation is also EGFR and MAPK dependent (Su et al., 2002; Fan and Sun, 2004; Park et al., 2004; Liang et al., 2005; Downs and Chen, 2010). Although there are four PKC isotypes in both CCs and oocytes, protein kinase C ε (PKCε) has been detected only in CCs (Downs et al., 2015). Thus, it is reasonable and interesting to evaluate the relationship between PKCε and MAPK in the context of Cx43 action in FSH-induced oocyte meiotic resumption.

In this study, a FSH-induced COC maturation model was designed to elucidate the following mechanisms: (1) the dynamic changes in Cx43 phosphorylation and cAMP level during FSH-induced meiotic resumption; (2) the possible involvement of PKCε in the upregulation of Cx43 phosphorylation and (3) the cross-talk between PKCε and MAPK.

## RESULTS

### Dynamic changes in cAMP and pCx43 levels in response to FSH

**Fig. 1.**
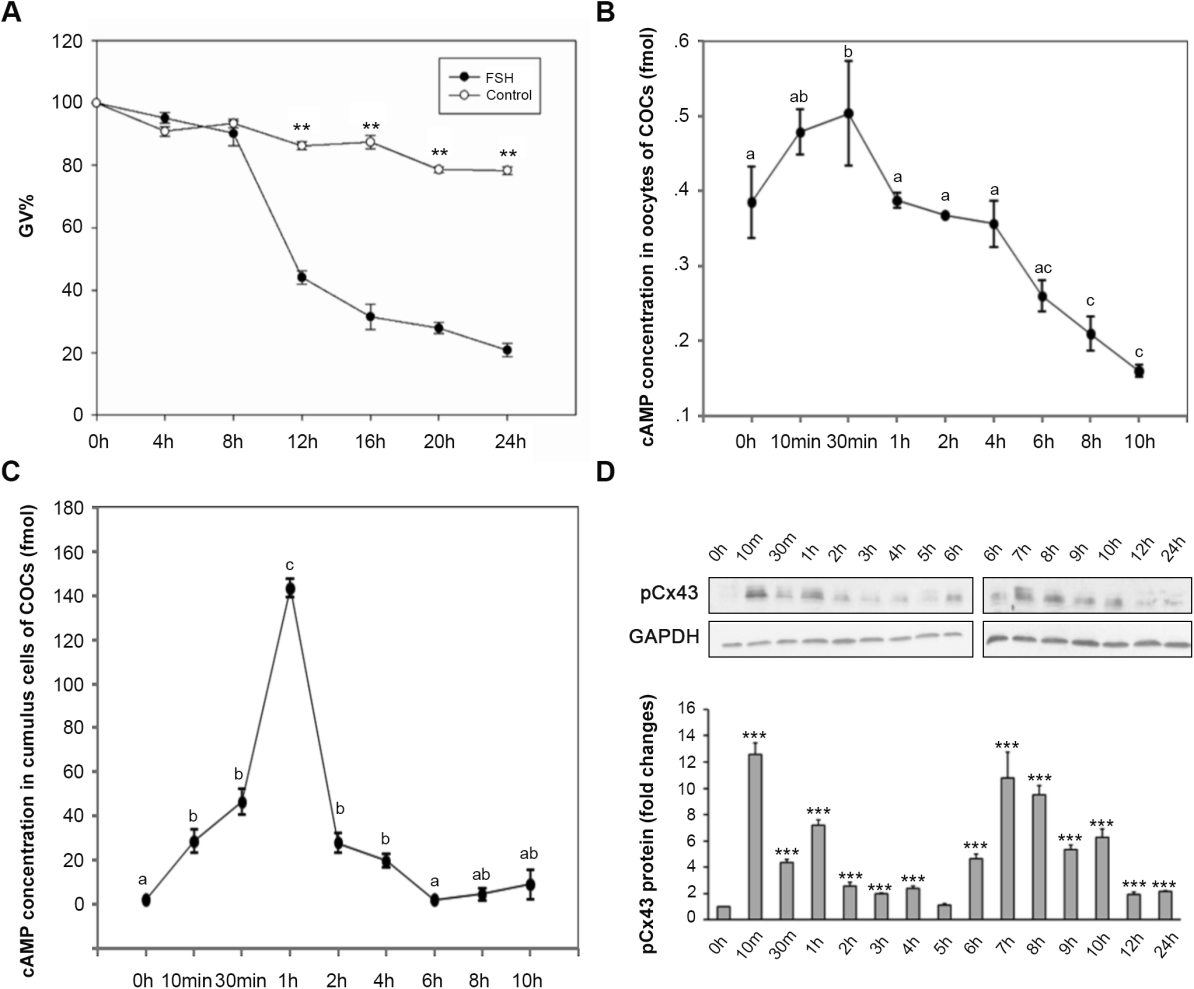
**The kinetic changes of cAMP levels in both oocyte and cumulus cells, and phosphorylation of Cx43 in FSH-induced mouse oocyte meiotic resumption.** (A-C) COCs were *in vitro* cultured with or without 0.05 IU/ml FSH in HX-media for 24 h (A) or with FSH for 10 h (B,C). (A) For evaluating the oocyte meiosis kinetic changes, the rate of GV stage oocyte was scored at 4 h intervals during the 24 h culture. Data was represented as mean percentage of GV±s.e.m. of three independent experiments. **P<0.01, compared with each corresponding TP in the control group. For detecting cAMP kinetic changes in response to FSH stimulation, oocytes (B) and cumulus cells (C) were respectively collected at various TPs during 10 h culture for RIA. The means±s.e.m. of the results of three individual experiments were presented, different letters indicate *P*<0.05 (*t*-test) to 0 h. (D) The effect of FSH on pCx43. Cumulus cells from 50 COCs treated with or without 0.05 IU/ml FSH in HX-media were collected at various TPs during 24 hs *in vitro* culture period for immunoblotting. The experiments were performed three times with similar results. *** indicates *P*<0.001 (*t*-test), compared to 0 h.

FSH induced the maturation of COCs cultured *in vitro*. When mouse COCs were cultured in hypoxanthine (HX) media for 24 h, over 80% of oocytes were sustained at the germinal vesicle (GV) stage, while approximately 78% of oocytes resumed meiosis in the FSH group (*P*<0.01) (Fig. 1A). These results are in accordance with those of previous reports (Su et al., 2002; Byskov et al., 2015). We noticed that a significant deviation occurred at the 8 h time point (TP). By the 12 h TP, oocytes at the GV stage in the FSH group became significantly different from the control group; only 44.6% of oocytes were sustained at the GV stage in the FSH group, but approximately 86.3% of oocytes were sustained at the GV stage in the control group (*P*<0.01).

There were cAMP surges in both oocytes and CCs during FSH-induced COC maturation, but the peak levels of cAMP in the oocytes (Fig. 1B) and CCs (Fig. 1C) during the first 10 h of the *in vitro* maturation (IVM) period was time dependent. In oocytes, the cAMP level rose slightly during the first 30 min culture period (0 h: 0.3532±0.0267 fmol per oocyte versus 10 min: 0.4639±0.0272 fmol per oocyte) (*P*<0.05). After that, the cAMP level in oocytes decreased steadily. At the 8 h TP, the cAMP level in the FSH group was much lower than that in the 0 h TP (8 h: 0.2401±0.0200 fmol per oocyte versus 0 h: 0.3532±0.0267 fmol per oocyte) (*P*<0.05). In CCs, however, although the cAMP level increased significantly from the 10 min TP on (0 h: 2.8686±1.0112 fmol versus 10 min: 33.7474±6.1030 fmol) (*P*<0.05), the most significant increase occurred at the 1 h TP; this level was more than 70-fold higher than that at 0 h in the same group (0 h: 2.8686±1.0112 fmol versus 1 h: 151.6914±8.7839 fmol) (*P*<0.01). After that, the cAMP level decreased dramatically (1 h: 151.6914±8.7839 fmol versus 2 h: 29.3154±3.5448 fmol) (*P*<0.01), until it resumed a level similar to that of the original state at the 6 h TP (6 h: 3.7659±1.9155 fmol versus 0 h: 2.8686±1.0112 fmol) (*P*>0.05).

Western blot (WB) analysis showed that with the FSH stimulation, obvious Cx43 phosphorylation occurred twice in the CCs of COCs during the first 12 h of a 24 h culture *in vitro* (Fig. 1D). The first phosphorylation occurred at a time corresponding with the cAMP surge (10 min TP), while the second phosphorylation occurred during the meiosis pre-initiation period (6 h TP–8 h TP). The results indicate that the cAMP level changes in both the oocytes and CCs, as well as the phosphorylation of Cx43, take part in FSH-induced oocyte meiotic resumption *in vitro*.

### PKCε is involved in FSH-induced Cx43 phosphorylation

**Fig. 2.**
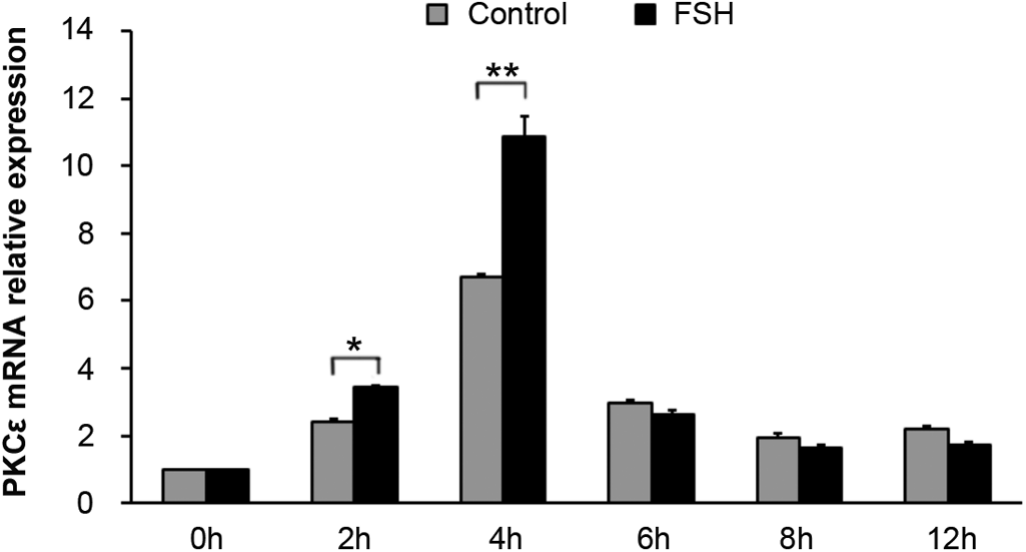
**The expression pattern of PKCε mRNA within cumulus cells of COCs during FSH-induced oocyte meiotic resumption.** COCs were treated with 0.05 IU/ml FSH in HX-media *in vitro*. COCs cultured in HX-medium were used as a control. Samples of cumulus cells at various TPs were collected for real-time RT-PCR. PKCε mRNA levels induced by FSH were analyzed. * indicates *P<*0.05 and ** indicates *P*<0.01, compared to the controls of the same group, respectively. The experiments were performed three times with similar results.

Real-time PCR analysis showed a significant enhancement in the *PKCε* mRNA in the CCs of COCs from the 2 h TP–4 h TP in response to FSH induction compared with the control (Fig. 2) (*P*<0.01). This result suggests that PKCε is involved in COC maturation during FSH induction.

**Fig. 3.**
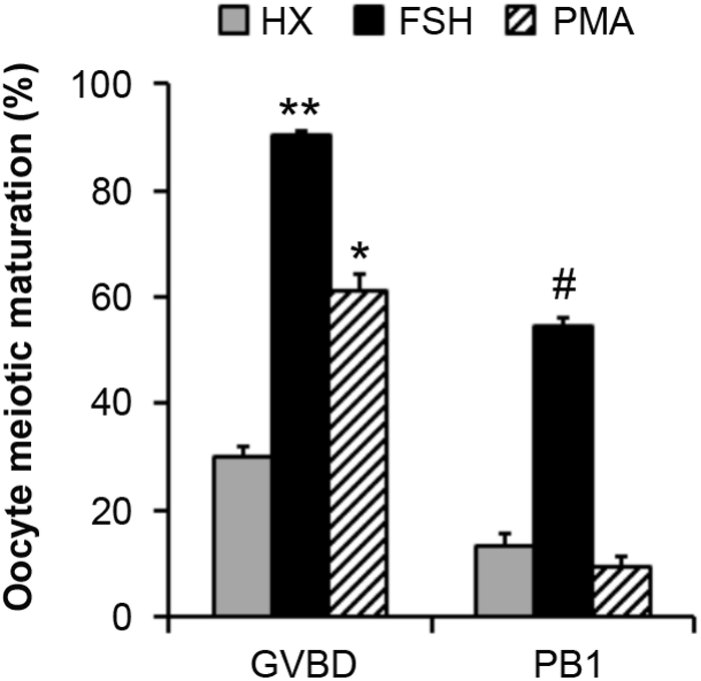
**The effect of PMA on the meiotic resumption of mouse COCs.** COCs were *in vitro* cultured in HX-media supplemented with 0.05 IU/ml FSH or 16.2 nM PMA for 24 h. The GVBD% and PB1% were recorded after culture. COCs cultured in HX-media were used as a control. * indicates *P<*0.05 and ** indicates *P*<0.01, compared to the controls of the same group, respectively. # indicates *P<*0.05, compared to the controls of the same group, respectively. The experiments were performed three times with similar results.

To evaluate the action of PKC in mouse oocyte meiotic resumption, the effect of a PKC agonist phorbol myristate acetate (PMA) on COC maturation was compared with that of FSH. The results showed that after 24 h of culture, FSH significantly induced COC maturation, as judged by the ratios of oocytes progressing to the germinal vesicle breakdown (GVBD) (90.47% versus 30.13%) (*P*<0.01) and first polar body (PB1) stages (54.62% versus 12.81%) (*P*<0.05). PMA significantly increased the GVBD ratio of COCs (61.72% versus 30.13%) (*P*<0.05), which partially simulated the role of FSH (61.72% and 90.47%) (*P*<0.05). However, it had no obvious effect on improving the PB1 ratio (9.34% versus 12.81%) (*P*>0.05) (Fig. 3).

**Fig. 4.**
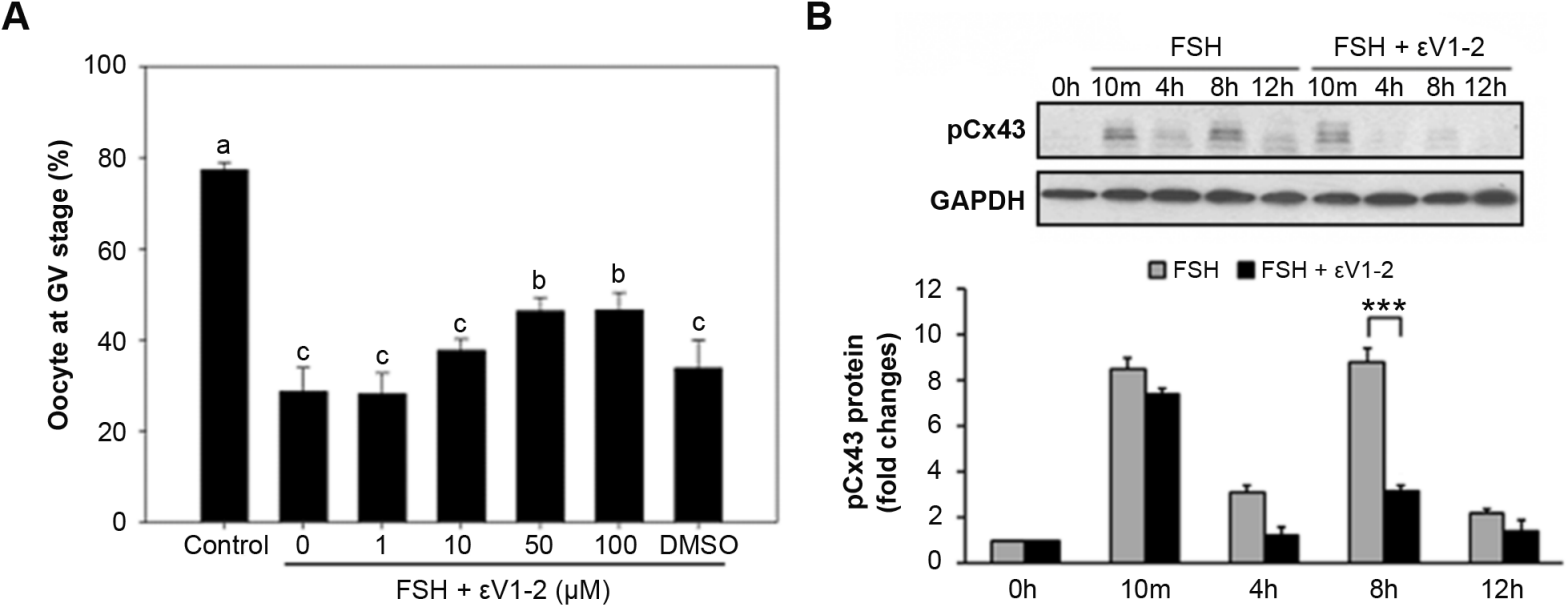
**The effect of PKCε specific inhibitor on both FSH-induced meiotic resumption and Cx43 phosphorylation in cumulus cells of COCs.** (A) When COCs were *in vitro* cultured, different concentrations of specific inhibitor PKCε and εV1-2 (1-100 μM) were respectively added into HX-media containing 0.05 IU/ml FSH. The percentage of GV was recorded at 24 h of culture. Different letters on each column indicate *P<*0.05 compared to the control, respectively. (B) Cumulus cells from 50 COCs treated with or without 50 μM εV1-2 in HX-media containing 0.05 IU/ml FSH were collected at various TPs for immunoblotting. The experiments of each assay were performed three times with similar results. *** indicates *P*<0.001 compared to the control of the same group.

**Fig. 5.**
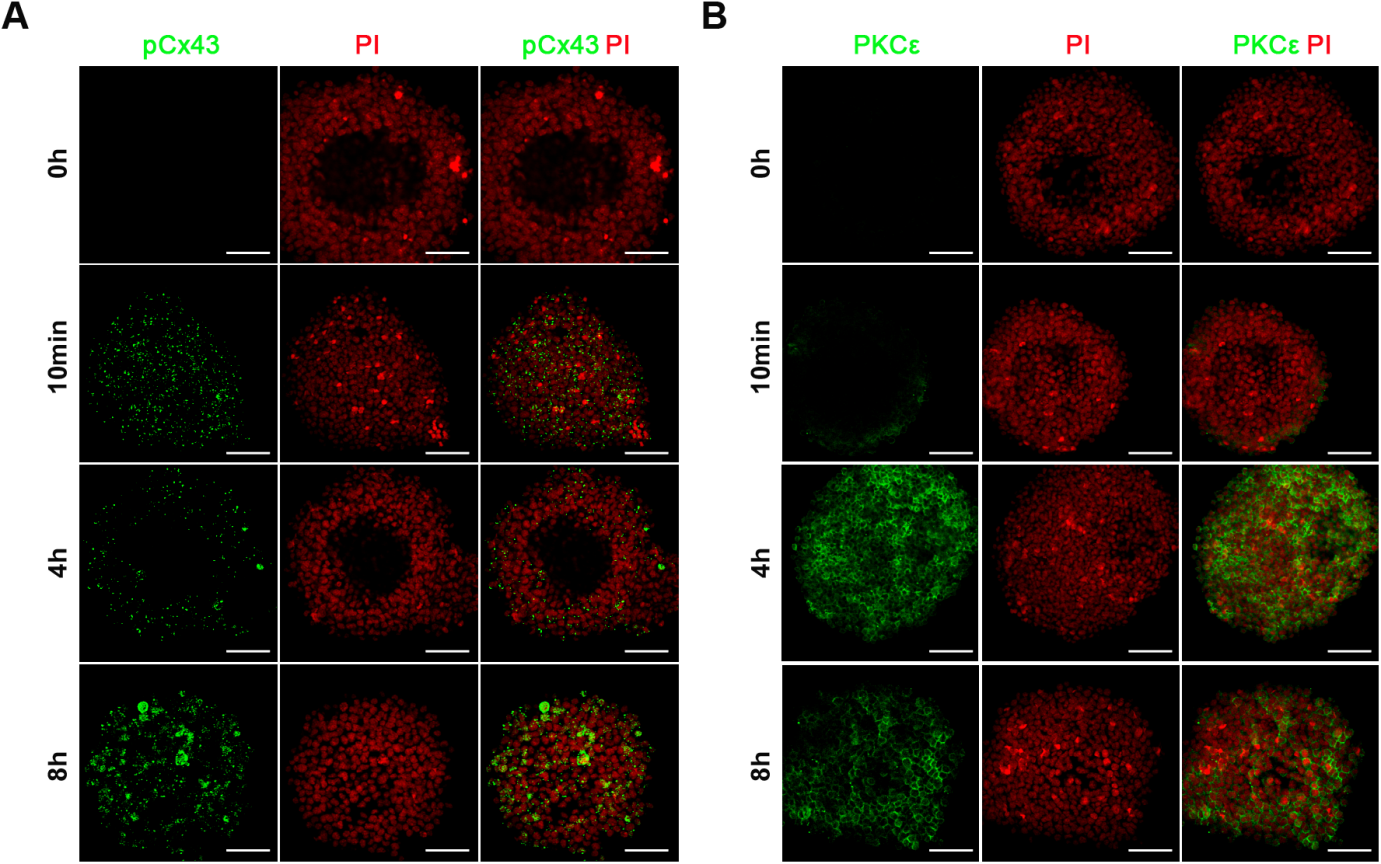
**Immunofluorescence localization of pCx43 and PKCε in mouse COCs.** (A-B) pCx43 (A) and PKCε (B) immunostaining (green) in cumulus cells of COCs were performed at various TPs after FSH induction. Nuclei were dyed with propidium iodide (PI, red). Scale Bars: 50 μm. The experiments were performed three times with similar results.

To study the function of PKCε specifically in CCs after FSH induction, myr-PKCε V1-2 (εV1-2), a specific inhibitor of PKCε, was added to the culture media. The results showed that after 24 h of culture, 50 μM and 100 μM of εV1-2 significantly inhibited FSH actions in oocyte meiosis; the GV ratios were 46.37% and 46.55%, respectively, while that in the FSH group was 28.67% (*P*<0.05) (Fig. 4A). Meanwhile, the action of PKCε on pCx43 was also confirmed. However, 50 μM of εV1-2 had no obvious effect on Cx43 phosphorylation, while it significantly inhibited the second phosphorylation of Cx43 (8 h TP) (*P*<0.001) (Fig. 4B). Accordingly, the immunofluorescence results in CCs showed pCx43 expression at the 10 min, 4 h and 8 h TPs after FSH induction (Fig. 5A). Although the relative expression of PKCε in CCs was low before the 4 h TP (Fig. 5B), it showed significantly higher expression in CCs after the 4 h TP during FSH induction (Fig. 5B). These results indicate that PKCε is involved in the second Cx43 phosphorylation during FSH induction.

### PKCε functions downstream of MAPK signaling in FSH-induced COC IVM

**Fig. 6.**
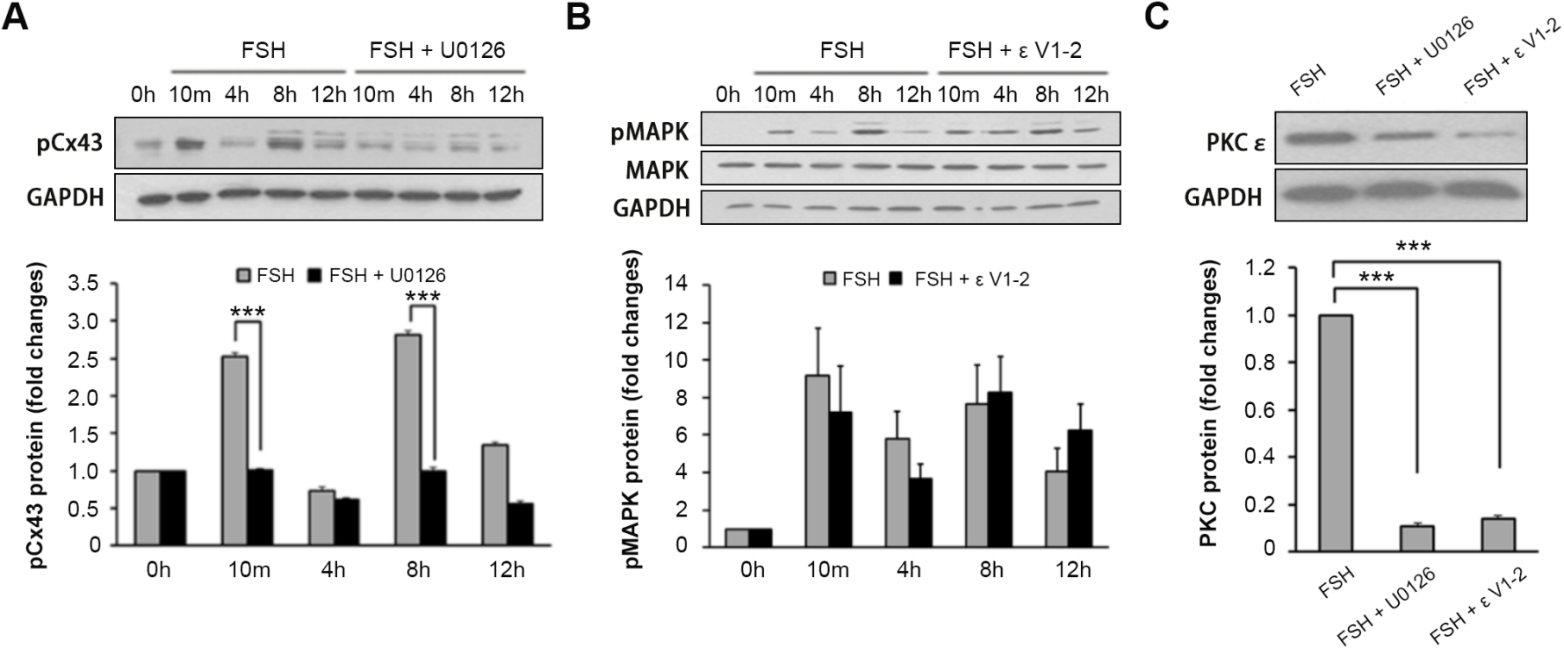
**The correlation between PKCε and MAPK in FSH-induced oocyte meiotic resumption.** (A) The effect of MAPK inhibitor U0126 on Cx43 phosphorylation. COCs treated with or without 10 μM U0126 in HX medium containing 0.05 IU/ml FSH were collected at various TPs for immunoblotting. *** indicates *P*<0.001 compared to the control of the same group. (B) The effect of PKCε inhibitor εV1-2 on MAPK phosphorylation. Cumulus cells from 50 COCs treated with or without 50 μM εV1-2 in HX medium containing 0.05 IU/ml FSH were collected at various TPs for immunoblotting. (C) The effect of U0126 on PKCε translocation in response to FSH stimulation. COCs were cultured in HX medium containing 0.05 IU/ml FSH, 10 μM U0126 and 50 μM εV1-2 (negative control) were used. Cumulus cells from 50 COCs were collected at 8 h for immunoblotting. The experiments were performed three times with similar results. *** indicates *P*<0.001 compared to the FSH group.

To clarify the relationship between MAPK and PKCε in the context of oocyte meiotic resumption in the FSH-induced IVM model, the mutual actions of MAPK and PKCε were studied using their respective inhibitors, U0126 at 10 μM and εV1-2 at 50 μM. The results showed that MAPK inhibition notably inhibited the two Cx43 phosphorylations stimulated by FSH (Fig. 6A) (*P*<0.001) and εV1-2 had no effect on FSH-induced MAPK activation in CCs (*P*>0.05) (Fig. 6B), suggesting that MAPK unlikely acts downstream of PKCε. In contrast, MAPK inhibition decreased PKCε expression in CCs dramatically (*P*<0.001) (Fig. 6C), indicating that MAPK is involved in the activation of PKCε. These results suggest that PKCε is a downstream signal of MAPK and that both of them are involved in the Cx43 phosphorylation induced by FSH in CCs *in vitro*.

**Fig. 7.**
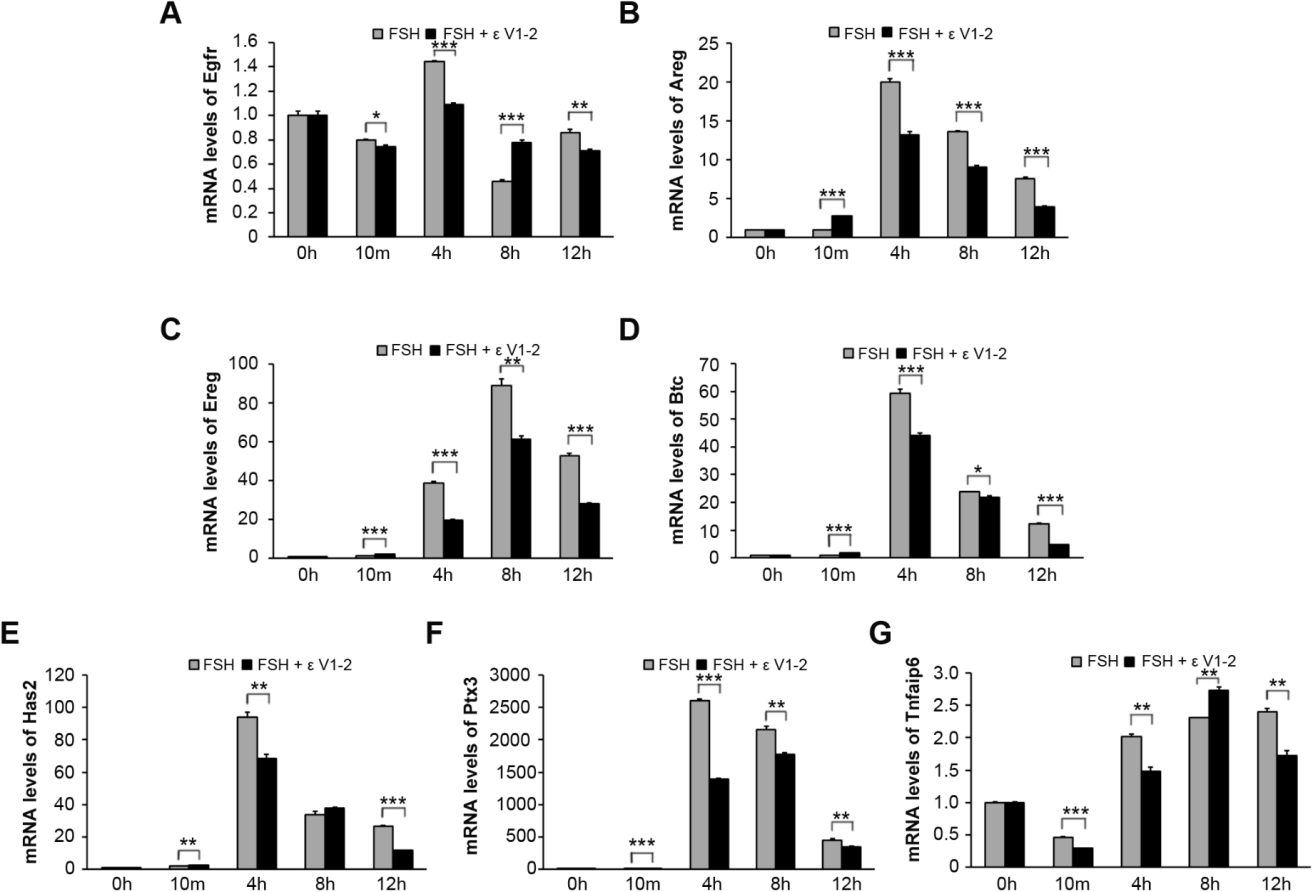
**The expression patterns of serial genes related to oocyte meiosis resumption and cumulus expansion.** (A-G) The mRNA levels of *Egfr*, *Areg*, *Ereg* and *Btc* (A-D, respectively), which are related to oocyte meiotic resumption and *Has2*, *Ptx3* and *Tnfaip6* (E-G, respectively)*,* which are cumulus expansion related, were examined through real time PCR. * indicates *P<*0.05, ** indicates *P*<0.01, *** indicates *P*<0.001, compared to the controls of the same group.

To further confirm that PKCε is pivotal for mediating the maturation of COCs in response to FSH, the expression patterns of a series of genes related to oocyte meiotic resumption (*Egfr*, *Areg*, *Ereg* and *Btc*) and cumulus expansion (*Has2*, *Ptx3* and *Tnfaip6*) were studied through real-time PCR. The results showed that from the 4 h TP on, all of the genes examined were elevated in response to FSH stimulation (Fig. 7). However, whenever PKCε activity was blocked, the mRNA levels of these genes were downregulated accordingly (Fig. 7). These results confirm that PKCε is important for COC maturation in response to FSH *in vitro*.

## DISCUSSION

**Fig. 8.**
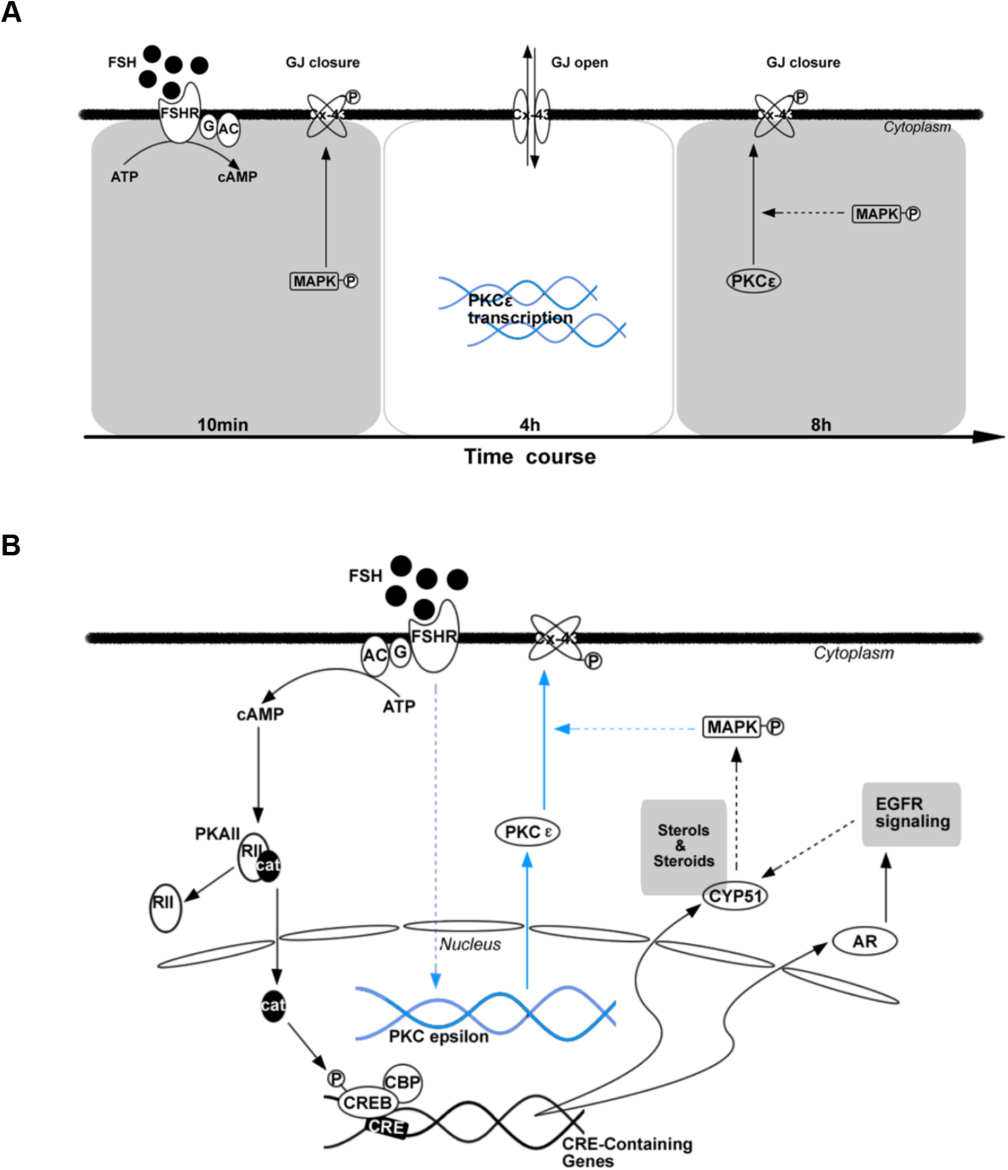
**The mechanism of protein phosphorylation as well as regulation of Cx43 during FSH-induced mouse oocyte maturation.** (A-B) In *in vitro* cultured COCs model, there exists two Cx43 phosphorylations responding to FSH induction. (A) The MAPK may take part in both process and plays a key role in the second Cx43 phosphorylation, while PKCε is only involved in the second Cx43 phosphorylation. PKCε may be a downstream molecule of MAPK signaling in response to FSH induction *in vitro* (B).

The present study investigated the dynamic change in and potential mechanism of Cx43 phosphorylation during FSH-induced meiotic resumption. The results indicate that two Cx43 phosphorylations occur during the FSH induction process. A correlation between the reduced cAMP level within oocytes and the second Cx43 phosphorylation during FSH-induced maturation was observed. FSH upregulated PKCε expression in the CCs. Importantly, MAPK activation was involved in the first transient Cx43 phosphorylation in CCs and played a key role in the second Cx43 phosphorylation induced by FSH, while PKCε was only involved in the second Cx43 phosphorylation in CCs. These findings indicate that PKCε is a downstream protein of the MAPK signaling pathway. Thus, a general mechanism of Cx43 phosphorylation and regulation (Fig. 8A) during FSH-induced mouse oocyte maturation, as well as a possible signaling pathway, is presented (Fig. 8B).

At the time of ovulation, GJC between an oocyte and GCs is attenuated, which in turn reduces the cGMP level in the oocyte, thus allowing it to complete meiosis (Winterhager and Kidder, 2015). Inhibition of the catalytic activity of phosphodiesterase 3A (PDE3A), an oocyte-specific phosphodiesterase, is vital for sustaining a high level of cAMP before the LH surge (Norris et al., 2009; Vaccari et al., 2009). PDE3A is inhibited by cGMP, which diffuses from the GCs into the oocyte through GJs (Norris et al., 2009). *In vitro*, treating COCs with FSH for 1 h can induce a 100-fold increase in the cAMP level in CCs and a two to three fold increase in oocytes (Salustri et al., 1985; Sanford and Batten, 1989), which is in accordance with this research. The peak value of cAMP within CCs may be related to the gonadotropin-mediated activation of cAMP-dependent protein kinase (PKA) II that initiates meiotic signaling (Hunzickerdunn, 1981; Ning et al., 2008). In contrast, the small increase in the oocyte cAMP level detected in this study may contribute to sustaining meiotic arrest (Conti et al., 2012).

Follicular somatic cells, the ooplasm and GJC are all important factors that affect oocyte developmental competence (Thomas et al., 2004). Cx43 is believed to be important for oocyte maturation because it has a particularly long C-terminal tail that governs and regulates pH gating, channel assembly and plaque size (Laird, 2014). In humans, the level of Cx43 expression is positively correlated with the strength of intercellular coupling among CCs, demonstrating that Cx43 is a major contributor to GJs (Winterhager and Kidder, 2015). Similarly, Cx43 knockout mouse fetuses exhibited severe reproduction problems, with folliculogenesis arrested at the early antral stage and a reduced number of germ cells in both sexes during early gestation; however, the remaining few oocytes could initiate follicle formation (Juneja et al., 1999). Interestingly, the results from this study indicate that there are two phosphorylations of Cx43 in FSH-induced COC maturation. We speculate that the first phosphorylation of Cx43 accounts for a dramatic increase in cAMP level in CCs, which does not result in a similar amplitude increase of cAMP in oocytes. During this time, pCx43 appears as quickly as 10 min after FSH induction, which may result in closure of the GJs between the CCs and oocytes (Cooper et al., 2000). However, the second phosphorylation of Cx43 occurs just before the initiation of oocyte meiosis *in vitro*, which is in accordance with the time at which cAMP decreases within an oocyte and the oocyte starts to resume meiosis significantly. Since little cAMP accumulates in the follicle prior to LH stimulation, it is likely that cGMP, rather than cAMP, may be the diffusing molecule critical for maintaining meiotic arrest (Liang et al., 2005; Norris et al., 2009; Zhang et al., 2010).

*In vivo*, in response to LH, MAPK cascades mediate the phosphorylation of Cx43 and thus induce a decrease in the cAMP and cGMP levels in oocytes and subsequent meiosis reinitiation (Norris et al., 2008, 2010; Conti et al., 2012). LH-dependent MAPK activation occurred downstream of cAMP and was dependent on PKA activation (Juneja et al., 1999). PKC pathways also mediate MAPK activation (Woods and Johnson, 2007) and simulate FSH action (Fan et al., 2004), which is consistent with our conclusion. For CC-specific PKCε expression (Downs and Chen, 2010), we noticed a significant increase in response to FSH. However, unlike MAPK (Su et al., 2002) and EGFR inhibitors (Downs and Chen, 2010), inhibition of PKCε did not achieve a similar effect in stopping FSH-induced oocyte maturation, which suggests that the PKCε pathway is only a part of the FSH-induced signal transductions in mouse COC IVM. In line with this, we found that U0126 suppressed both Cx43 phosphorylations (10 min and 8 h TPs), while V1-2 inhibited only the second phosphorylation of Cx43 *in vitro*. PKCε is involved in the second phosphorylation of Cx43 in response to FSH induction because it helps to stop the transportation of key molecules through GJs before oocytes resume meiosis (Ning et al., 2008). The reason why PKCε did not take part in the first phosphorylation of Cx43 needs further study.

Regarding the relationship between MAPK and PKCε, we have found that inhibition of MAPK could notably inhibit the two Cx43 phosphorylations stimulated by FSH, indicating that both events participate in the process, which is in agreement with the findings of previous reports (Sela-Abramovich et al., 2005; Norris et al., 2008). Although a previous study has indicated that MAPK might be downstream of PKC in CCs stimulated by PMA (Fan and Sun, 2004), our results support the hypothesis that PKCε is a downstream signal of MAPK and that both of them are involved in the phosphorylation of Cx43 induced by FSH in CCs *in vitro*. Alternatively, we have demonstrated that inhibition of PKCε has no effect on FSH-induced MAPK phosphorylation in CCs, suggesting that MAPK unlikely acts downstream of PKCε. Instead, inhibition of MAPK decreased PKCε expression in CCs, indicating that MAPK is involved in the activation of PKCε.

In conclusion, this study shows that Cx43 is phosphorylated twice in the process of FSH-induced COC IVM and that the second phosphorylation might be related to the decrease in cAMP level in oocytes for initiating meiosis. PKCε responds to FSH induction and might be a downstream molecule of the MAPK signaling pathway; in addition, it is required for the second phosphorylation of Cx43.

## MATERIALS AND METHODS

### Animals

Mice were obtained from the Laboratory Animal Center of the Institute of Genetics and Developmental Biology (Beijing, China). White female Kunming mice (KM, outbreed strain) at 21-23 days old were used for all experiments. The mice were housed under controlled temperature (24-26°C) and lighting (12 h light/12 h darkness) conditions with food and water available *ad libitum*. Follicle development was primed in each mouse by the intraperitoneal injection of 5 U equine chorionic gonadotropin (eCG). The mice were euthanized by cervical dislocation 44-48 h later. The protocols for animal use conformed to the guidelines and regulatory standards of the Institutional Animal Care and Use Committee of China Agricultural University. All experiments were approved by the Institutional Animal Care and Use Committee of China Agricultural University (license number: SKLAB-2014-01-17).

### Chemicals

All reagents and chemicals used in this study were obtained from Sigma-Aldrich Corp., unless otherwise indicated. FSH was prepared as stock solutions in distilled PBS containing 0.1% BSA, and the final concentration used for culture was 0.05 IU/ml. eCG (Sansheng Pharmaceutical Co. Ltd, Ningbo, China) was dissolved (1000 U) in 20 ml of 0.9% sodium chloride, sub-packed into 1 ml centrifuge tubes at 50 U/ml, and stored at −20°C before use. Polyclonal rabbit anti-Cx43 antibody (Ser-279/Ser-282), monoclonal rabbit anti-PKCε antibody, monoclonal mouse anti-GAPDH antibody, and relative HRP-conjugated secondary antibodies were purchased from Santa Cruz Biotechnology Inc. Monoclonal mouse anti-MAPK-activated antibody (phosphorylated ERK1 and 2) was purchased from Cell Signaling Technology. The PKCε inhibitor εV1-2 was purchased from Biomol GmbH Waidmannstr. (Hamburg, Germany) and dissolved in dimethyl sulfoxide (DMSO) before use, of which the maximum working concentration was 100 μM. U0126 was prepared at 50 mM in DMSO. The final concentration of DMSO was less than 0.1%, which had no significant effect on oocyte maturation (data not shown). These stock solutions were further diluted with culture medium before use. The test concentrations of these reagents were in accordance with previous reports. The culture medium used for this study was M199 (Invitrogen, Carlsbad, CA, USA) medium containing 4 mM HX, 0.23 mM sodium pyruvate, 2 mM glutamine, 3 mg/ml lyophilized crystallized BSA, 75 mg/ml potassium penicillin G and 50 mg/ml streptomycin sulfate.

### COCs isolation and IVM

COCs were isolated from the ovaries of 23- to 25-day-old female mice primed with eCG as described above, and incubated in culture medium with HX to prevent spontaneous oocyte maturation until distribution into experimental groups. After being washed in HX-supplemented medium, groups of approximately 50 COCs were cultured for as long as 24 h in 2 ml of culture media supplemented with or without 0.1 IU/ml FSH. The COCs were cultured at 37°C in an atmosphere of 5% CO_2_ and 95% air with saturated humidity.

For the *in vitro* culture of COCs, each experiment was repeated at least three times. At the termination of culture, oocyte maturation was assessed by scoring released oocytes for GVs, GVBD or PB1 after CC removal. Then, all samples were immediately frozen in liquid nitrogen and stored at −80°C until analyzed for mRNA or protein levels, as described below. Alternatively, for PKCε and pCx43 localization, COCs were fixed for immunofluorescence without removing the CCs.

### Radioimmunoassay (RIA) of cAMP levels

After *in vitro* culture, the CCs were removed from the oocytes, and either the CCs or oocytes were transferred in less than 5 μl of media into 100 μl of 0.1 M HCl. The cAMP level within either the CCs or oocytes was measured using a specific radioimmunity kit (Beckman Coulter Inc., Fullerton, CA, USA) according to the protocols provided by the manufacturer.

### RNA isolation and real-time PCR

Total RNA of CCs isolated from *in vitro* cultured COCs was extracted with TRIzol Reagent (Invitrogen) according to the manufacturer's protocol. The quantity and quality of the total RNA were determined using a NanoDrop system (Thermo Fisher Scientific). Reverse transcription was conducted with oligo (dT) primers using Moloney murine leukemia virus reverse transcriptase according to the manufacturer's instructions (Promega, Madison, WI, USA). Gene expression changes were analyzed by real-time PCR in 96-well plates (Applied Biosystems, Life Technologies) in 15-μl reaction volumes and were normalized to β-actin. PCR was performed on an ABI 7500 Real-Time PCR System (Applied Biosystems). Gene expression levels from each experiment are presented as changes relative to a specific group (control) whose expression level was set at one. Each experiment was repeated independently three times. All primers for real-time PCR are listed in Table S1.

### Immunofluorescence

COCs that were cultured *in vitro* for various durations were fixed for 10 min at room temperature in 4% paraformaldehyde. For the convenience of comparing the results of different samples at the same TP, the following immunofluorescence procedures were performed for all COCs. COCs were washed in 1% BSA three times, treated with 1% Triton X-100 at 37°C for 30 min and blocked with 10% (v/v) normal goat serum overnight. Then, the COCs were incubated with the primary antibody (rabbit anti-PKCε monoclonal antibody or rabbit anti-pCx43 polyclonal antibody at a ratio of 1:100) at 4°C overnight. Subsequently, the COCs were washed with PBS and incubated with FITC-labeled goat anti-rabbit antibody for 1 h at room temperature. After being washed, the DNA in the cells was labeled with 10 μg/ml propidium iodide (PI) for 1 min. The COCs were then placed onto the center points of slides, where anti-fluorescence quenching agents were preloaded. The samples were then stamped with slides that were preloaded with wax at each of the four corners (Vaseline: paraffin, 9:1).

### Electrophoresis and WB analyses

The CC proteins from 50 COCs per sample were subjected to WB according to previously reported procedures (Ning et al., 2008). In brief, after culture, proteins were extracted with double-strength electrophoresis sample buffer composed of 125 mM Tris, pH 6.8, 4% (w/v) SDS, 20% (w/v) glycerol, 10% (v/v) β-mercaptoethanol (β-ME) and 0.004% (w/v) bromophenol blue, supplemented with 1 mM phenylmethyl sulfonyl fluoride and 1 mM sodium orthovanadate for 20 min on ice, and stored at −80°C. The PKCε membrane proteins were prepared using a Membrane Extraction Kit according to manufacturer's instructions (Applygen Technologies Inc., Beijing, China). Before electrophoresis, the lysates were heated to 100°C for 5 min, immediately cooled on ice, and then centrifuged at 12,000 ***g*** for 5 min. The proteins were separated by SDS-PAGE with a 4% stacking gel and a 10% separating gel for 50 min at 160 V and were then electrically transferred to a nitrocellulose membrane (Amersham Pharmacia Biotech, Braunschweig, Germany). The membrane was saturated with 5% nonfat dry milk and then incubated with corresponding antibody. Proteins were detected using a SuperSignal West Pico (enhanced chemiluminescence) detection system (Pierce Chemical Co., Rockford, IL, USA). For reprobing total MAPK, the blots were stripped of the bound antibodies by being washed in a stripping buffer (100 mM β-ME; 20% sodium dodecyl sulfate; 62.5 mM Tris, pH 6.7) and then reprobed with anti-MAPK antibody. Blot density was measured using AlphaEaseFC Software if needed.

### Statistical analyses

All experiments were performed at least three times, and the values are given as the mean±s.e.m., with each experiment performed in triplicate. Data were analyzed by *t*-test or ANOVA using StatView software (SAS Institute Inc., Cary, NC, USA). A *P* value of less than 0.05 was considered statistically significant.

## Supplementary Material

Supplementary information
