# Supplementary material for: Involvement of PKCε in FSH-induced connexin43 phosphorylation and oocyte maturation in mouse
Source: Biol Open. 2018 Jul 30;7(8):bio034678. doi: 10.1242/bio.034678 (PMC6124567; doi:10.1242/bio.034678)
Supplement: Supplementary information [file biolopen-7-034678-s1.pdf]

Biology Open (2018): doi:10.1242/bio.034678: Supplementary information

Table S1

The primers used for real-time PCR

Genes	Forwards (5' to 3')	Reverses (5' to 3')
PKCε	ACTGCACCATCCAGTTCGAG	CTTCACCCGATGATCCCGAG
EGFR	CTTCACCCGATGATCCCGAG	GGTGTGAGAGGTTCCACGAG
AREG	GGTCTTAGGCTCAGGCCATTA	CGCTTATGGTGGAAACCTCTC
EREG	GCATCCCAGGAGAATCCGAG	ACATCGCAGACCAGTGTAGC
BTC	AAACCCACTTCTCTCGGTGC	AAACAGGTCCACTCGCTCAC
HAS2	TGGTGAGACAGAAGAGTCCCA	TGGTGAGACAGAAGAGTCCCA
PTX3	CTGTGCTGGAGGAACTGCG	CAGGATGCACGCTTCCAAAA
Tnfaip6	AATCCGGCTCAACAGGAGTG	AGCAGACCTGGTTGTCATCG
β-actin	CTTTGCAGCTCCTTCGTTGC	CCTTCTGACCCATTCCCACC

Biology Open • Supplementary information
